# Prognostic accuracy of the Hamilton Early Warning Score (HEWS) and the National Early Warning Score 2 (NEWS2) among hospitalized patients assessed by a rapid response team

**DOI:** 10.1186/s13054-019-2355-3

**Published:** 2019-02-21

**Authors:** Shannon M. Fernando, Alison E. Fox-Robichaud, Bram Rochwerg, Pierre Cardinal, Andrew J. E. Seely, Jeffrey J. Perry, Daniel I. McIsaac, Alexandre Tran, Steven Skitch, Benjamin Tam, Michael Hickey, Peter M. Reardon, Peter Tanuseputro, Kwadwo Kyeremanteng

**Affiliations:** 1 Division of Critical Care, Department of Medicine, University of Ottawa, Ottawa, ON Canada; 2 Department of Emergency Medicine, University of Ottawa, Ottawa, ON K1Y 4E9 Canada; 3 Division of Critical Care, Department of Medicine, McMaster University, Hamilton, ON Canada; 4 Department of Health Research Methods, Evidence, and Impact, McMaster University, Hamilton, ON Canada; 5 School of Epidemiology and Public Health, University of Ottawa, Ottawa, ON Canada; 6 Clinical Epidemiology Program, Ottawa Hospital Research Institute, Ottawa, ON Canada; 7 Department of Surgery, University of Ottawa, Ottawa, ON Canada; 8 Department of Anesthesiology and Pain Medicine, University of Ottawa, Ottawa, ON Canada; 9 Division of Emergency Medicine, Department of Medicine, McMaster University, Hamilton, ON Canada; 10 Division of Palliative Care, Department of Medicine, University of Ottawa, Ottawa, ON Canada; 11 Institut du Savoir Montfort, Ottawa, ON Canada

**Keywords:** Early warning systems, Rapid response teams, Intensive care unit, Sepsis

## Abstract

**Background:**

Rapid response teams (RRTs) respond to hospitalized patients experiencing clinical deterioration and help determine subsequent management and disposition. We sought to evaluate and compare the prognostic accuracy of the Hamilton Early Warning Score (HEWS) and the National Early Warning Score 2 (NEWS2) for prediction of in-hospital mortality following RRT activation. We secondarily evaluated a subgroup of patients with suspected infection.

**Methods:**

We retrospectively analyzed prospectively collected data (2012–2016) of consecutive RRT patients from two hospitals. The primary outcome was in-hospital mortality. We calculated the number needed to examine (NNE), which indicates the number of patients that need to be evaluated in order to detect one future death.

**Results:**

Five thousand four hundred ninety-one patients were included, of whom 1837 (33.5%) died in-hospital. Mean age was 67.4 years, and 51.6% were male. A HEWS above the low-risk threshold (≥ 5) had a sensitivity of 75.9% (95% confidence interval (CI) 73.9–77.9) and specificity of 67.6% (95% CI 66.1–69.1) for mortality, with a NNE of 1.84. A NEWS2 above the low-risk threshold (≥ 5) had a sensitivity of 84.5% (95% CI 82.8–86.2), and specificity of 49.0% (95% CI: 47.4–50.7), with a NNE of 2.20. The area under the receiver operating characteristic curve (AUROC) was 0.76 (95% CI 0.75–0.77) for HEWS and 0.72 (95% CI: 0.71–0.74) for NEWS2. Among suspected infection patients (*n* = 1708), AUROC for HEWS was 0.79 (95% CI 0.76–0.81) and for NEWS2, 0.75 (95% CI 0.73–0.78).

**Conclusions:**

The HEWS has comparable clinical accuracy to NEWS2 for prediction of in-hospital mortality among RRT patients.

**Electronic supplementary material:**

The online version of this article (10.1186/s13054-019-2355-3) contains supplementary material, which is available to authorized users.

## Introduction

Patients admitted to the hospital ward are at risk of short-term deterioration. Approximately 3–9% of hospitalized patients will demonstrate subjective or objective signs of clinical or biochemical deterioration during their hospitalization [1]. To improve quality of care among admitted patients, many hospitals have implemented rapid response teams (RRTs), which serve to identify and respond to deteriorating patients outside of the intensive care unit (ICU) [2]. While it remains unclear whether RRTs are associated with improved hospital mortality, the existing evidence does support their use in reducing cardiac arrests, facilitating ICU admission, and engaging patients and caregivers in discussions related to goals of care and end of life [3–7].

Early Warning Scores (EWSs), which incorporate objective measures of clinical status such as vital signs with clearly defined thresholds, serve to rapidly identify patients at risk of future deterioration (including death and ICU admission) [8]. A wide number of EWSs exist, and these scores have been found to have varying degrees of accuracy in predicting cardiac arrest and death [9, 10]. These tools may be utilized to increase monitoring, escalate care, or activate the RRT. RRTs may use these scores for the purposes of prognosis, in order to guide how aggressive to be with resuscitation, as well as disposition. Perhaps, the most well-known EWS, with the best prognostic characteristics, is the National Early Warning Score (NEWS), created by the Royal College of Physicians, London [11]. In 2017, the Royal College of Physicians updated the NEWS score (NEWS2), in order to improve precision (as based upon larger validation databases that included more varied patient types) [12] and to improve awareness of potential sepsis. A NEWS2 ≥ 5 represents the key threshold for urgent response.

The Hamilton Early Warning Score (HEWS) is a novel EWS that has been successfully pilot-tested in the emergency department (ED) setting at two hospitals [13, 14], and is being used in several centers in Canada. A HEWS ≥ 5 has been described as the threshold for concern of deterioration. Many similarities exist between the HEWS and the NEWS2 (Additional file 2: Figure S1), though little is known regarding how these two EWS compare. In particular, the HEWS and NEWS2 were designed to improve the early detection of all hospitalized patients at risk for deterioration, but particularly among those with suspected infection. Early identification, appropriate management, and disposition of patients with sepsis and septic shock are crucial to improving survival in this population [15]. Previous work investigating the use of the systemic inflammatory response syndrome (SIRS) criteria [16] and the quick Sequential Organ Failure Assessment (qSOFA) criteria [17, 18] in patients with suspected infection assessed by the RRT found both these tools to be unsatisfactory in predicting in-hospital mortality [19]. Given the variable accuracy of the existing EWSs in the RRT population, particularly among patients with suspected infection, new tools for risk stratification are required. The HEWS and NEWS2 are potential early warning tools that may be used in this setting; however, their prognostic accuracy among RRT patients is unknown. Therefore, we sought to compare the prognostic accuracy of the HEWS and the NEWS2 among patients assessed by the RRT, and in particular those patients with suspected infection.

## Materials and methods

Ethics approval for this study was obtained from The Ottawa Health Science Network Research Ethics Board.

### Study design, setting and subjects

We included adult patients from two individual academic hospitals within The Ottawa Hospital network (Ottawa, ON). This network has 1163 beds, and handles over 50,000 patient admissions, and 160,000 emergency department encounters annually. Each hospital has a combined medical-surgical ICU, with 28 ICU beds at each, and approximately 2500 combined ICU admissions per year. Approximately 3.9% of admitted patients per year will require RRT assessment. We retrospectively analyzed prospectively collected data between 2012 and 2016 from The Ottawa Hospital Data Warehouse, a health administrative database that has been widely used in previous research [20–22]. Data quality assessments were performed during development and are executed routinely as new data are included. Quality-assurance initiatives are conducted regularly to ensure completeness and accuracy.

At both participating hospitals, RRTs during daytime hours (approximately 0800–1700) are composed of an attending critical care physician, a registered nurse, and a respiratory therapist. Outside of these hours, a resident physician covers in-hospital with an on-call critical care physician available from home. The RRT calling criteria at the participating hospitals has been published previously (Additional file 1: Table S1) [23]. Outside of these objective indicators, healthcare providers are encouraged to activate the RRT for any reason of concern. The RRT responds only to inpatients, outpatients experiencing distress (e.g. in endoscopy or radiology suites), or patients requiring emergency care in hospital clinics or waiting rooms. The RRT does not respond to patients being assessed in the ED who have not yet been admitted to hospital.

We included all adult patients (≥ 18 years of age) who received RRT activation between May 1, 2012, and May 31, 2016. Cardiac arrests were excluded, as they involve activation of a different response team. Patients with multiple activations during their admission were categorized on the basis of their initial RRT activation only. Patients with “suspected infection” were defined as administration of oral or parenteral antibiotics and concomitant sampling of body fluid cultures [19]. This operational definition matches what was used in the Third International Consensus Definitions for Sepsis and Septic Shock (Sepsis-3) [17, 18]. We excluded patients with incomplete demographic or outcome data, as well as those for whom the HEWS or NEWS2 scores could not be calculated. We also excluded those patients with routine, scheduled RRT follow-up.

### Data collection

All data were obtained from The Ottawa Hospital Data Warehouse. For each patient, we abstracted basic demographic data, comorbidities, and Elixhauser Comorbidity Score [24]. At the time of admission, clerical staff collected demographic data, comorbidities, previous ED visits, previous hospital admissions, and previous ICU admissions in the year prior to the index admission. At the time of RRT assessment, the RRT nurse gathers and records data related to RRT activation. This includes the most recent vital signs and laboratory values at the time of RRT activation. Using these data, an investigator unaware of the patient’s clinical outcome calculated the HEWS and NEWS2 scores for each patient (Additional file 2: Figure S1). Outcome data was collected from hospital admission until either the point of discharge from hospital or in-hospital death. This included ICU length of stay (LOS), hospital LOS, and final disposition status.

The primary outcome was in-hospital mortality, comparing the HEWS and NEWS2 scores. We additionally evaluated the prognostic accuracy of HEWS and NEWS2 for prediction of ICU admission among RRT patients. In this secondary analysis, we excluded patients with limits of care that did not allow for ICU admission.

### Statistical analysis

We performed all statistical analyses with commercially available statistical packages (R, Version 3.3.3 and IBM SPSS, Version 24.0). Data are presented as either mean values, with standard deviation (SD), or medians, with interquartile range (IQR). The Student’s *t* test (parametric values), Mann-Whitney test (for non-parametric values), and χ^2^ (for categorical values) were used to determine between-group differences. To statistically compare relative accuracy between HEWS and NEWS2, we computed the relative true-positive rate (rTPR) and the relative false-positive rate (rFPR) [25, 26]. A rTPR is indicative of superiority in sensitivity of a test, and the rFPR is indicative of superiority in specificity of a test. In evaluating prediction of in-hospital mortality, we utilized logistic regression and calculated the area under the receiver operating characteristic curve (AUROC). In addition, we also calculated the number needed to evaluate (NNE) [27]. The NNE is the number of patients that is necessary to further evaluate or work-up in order to detect one outcome, and is the inverse of the positive predictive value. It has been argued that the NNE should be used instead of the AUROC in the evaluation of Early Warning Systems, given its ability to describe the trade-off between familiarity of activation and alarm fatigue [27]. A *P* value of ≤ 0.05 was considered statistically significant.

## Results

During the study period, the RRT was activated for 6132 discrete adult patients. Of these, 109 were excluded due to missing outcome data. An additional 532 patients (8.7%) did not have sufficient available data to calculate both the HEWS and NEWS2 scores and were therefore excluded (Additional file 3: Figure S2). This left 5491 patients for analysis. Of these patients, 1708 (31.1%) had suspected infection. Baseline characteristics of all patients are depicted in Table 1. Mean age was 67.4 years (SD, 16.3). The majority of these patients (3876, 70.6%) were admitted to hospital from home. The most common comorbidities noted were diabetes mellitus (45.1%), hypertension (33.7%), and arrhythmia (23.1%). Of note, 19.8% of patients had limits on the aggressiveness of their care which did not allow for cardiopulmonary resuscitation or ICU admission. A total of 1837 patients (33.4%) died during hospitalization. RRT call characteristics are displayed in Table 2. The majority of activations took place during daytime hours (64.3%). The most common reasons for RRT activation included respiratory distress (25.4%), altered level of consciousness (18.4%), and tachycardia or arrhythmia (18.1%).

**Table 1:** Baseline characteristics—entire cohort of RRT patients (*n* = 5491)

Variable	Value (*n* = 5491)
Age, years, mean (SD)	67.4 (16.3)
Male, *n* (%)	2834 (51.6)
Admission source, *n* (%)	
Home	3876 (70.6)
Acute care facility transfer	516 (9.4)
Long-term care facility t	588 (10.7)
Unknown	511 (9.3)
Comorbidities, *n* (%)	
Congestive heart failure	901 (16.4)
Arrhythmia	1268 (23.1)
Valvular disease	187 (3.4)
Peripheral vascular disease	374 (6.8)
Hypertension	1851 (33.7)
Chronic obstructive pulmonary disease	873 (15.9)
Diabetes mellitus	2476 (45.1)
Renal failure	566 (10.3)
Liver disease	323 (5.9)
Metastatic cancer	835 (15.2)
Elixhauser comorbidity score, mean (SD)	15.6 (8.8)
Emergency department visits in past year, median (IQR)	1 (0–3)
Hospital admissions in past year, median (IQR)	1 (0–2)
ICU admission in past year, *n* (%)	121 (2.2)
Limits of care, *n* (%)	
Full care	3750 (68.3)
ICU-level care, no CPR	373 (6.8)
Do Not Resuscitate	1087 (19.8)
Other/unknown	281 (5.1)

*CPR* cardiopulmonary resuscitation, *ICU* intensive care unit, *IQR* interquartile range, *SD* standard deviation

**Table 2:** RRT characteristics—entire cohort of RRT patients (*n* = 5491)

Variable	Value
Number of RRT activations during admission, median (IQR)	1 (1–1)
Time of initial RRT activation	
Daytime hours (0800–1659)	3530 (64.3)
Most recent vital signs	
Systolic blood pressure, mmHg, mean (SD)	141.4 (29.7)
Diastolic blood pressure, mmHg, mean (SD)	75.8 (14.1)
Mean arterial pressure, mmHg, mean (SD)	96.9 (17.1)
Heart rate, beats/min, mean (SD)	104.3 (29.2)
Temperature, degrees Celsius, mean (SD)	37.0 (0.9)
Oxygen saturation, %, median (IQR)	91.8 (7.8)
Most recent blood work	
White blood cell count, × 10^9^/L, median (IQR)	11.6 (7.2–16.3)
Hemoglobin, g/L, mean (SD)	102 (22.6)
Platelets, ×10^9^/L, mean (SD)	228.3 (1413)
Potassium, mmol/L, mean (SD)	4.3 (0.7)
Bilirubin, μmol/L, median (IQR)	11 (7–16)
Creatinine, μmol/L, median (IQR)	98 (64–165)
Urea, mmol/L, median (IQR)	2.4 (1.7–3.2)
Lactate, mmol/L, median (IQR)	1.8 (1.5–3.0)
Albumin, g/L, mean (SD)	25.9 (6.8)
INR, median (IQR)	1.1 (1.0–1.3)
Primary reason for RRT call, *n* (%)	
Respiratory distress	1395 (25.4)
Tachycardia/bradycardia/arrhythmia	994 (18.1)
Altered level of consciousness	1010 (18.4)
Hypotension	774 (14.1)
Hypertension	159 (2.9)
Airway concern	187 (3.4)
Seizure	71 (1.3)
Worried about patient	615 (11.2)
Other/unknown	286 (5.2)
Latency to first RRT activation from onset of concerning symptoms/signs	
< 1 h, *n* (%)	4131 (75.2)

*IQR* interquartile range, *RRT* rapid response team, *SD* standard deviation

Comparison of the prognostic accuracy of the HEWS and NEWS2 for prediction of in-hospital mortality among all RRT patients is displayed in Table 3. A HEWS ≥ 3 was found among 3181 patients (57.9%). A HEWS ≥ 3 had a sensitivity of 95.9% (95% CI 94.9–96.7) and a specificity of 44.6% (94.9–96.7). The NNE was 2.15 (95% CI 2.12–2.18). A HEWS ≥ 5 was found among 2578 patients (46.9%). Sensitivity was 75.9% (95% CI 73.9–77.9) and specificity was 67.6% (95% CI 66.1–69.1). The NNE was 1.85 (95% CI 1.81–1.89). In comparison, 3416 patients (62.2%) had a NEWS2 ≥ 5. The sensitivity of NEWS2 ≥ 5 was 84.5% (95% CI 82.8–86.2) and specificity was 49.0 (47.4–50.7). The NNE was 2.20 (2.16–2.25). The rTPR of NEWS2 to HEWS was 1.11 (95% CI 0.98–1.24, *P* = 0.09), while the rFPR was 1.57 (95% CI 1.46–1.69, *P* < 0.01). Prognostic accuracy of HEWS and NEWS2 for prediction of ICU admission among RRT patients is displayed in Additional file 4: Table S2. Sensitivity of HEWS ≥ 3 for prediction of ICU admission was 91.8% (95% CI 90.2–93.1), as compared to 83.4% (95% CI 81.4–85.3) for NEWS2. Specificity of HEWS ≥ 3 was 63.3% (95% CI 61.6–65.1), as compared to 64.5% (95% CI 62.7–66.2) for NEWS2.

**Table 3:** Prognostic accuracy of HEWS and NEWS2 for in-hospital mortality—entire cohort of RRT patients (*n* = 5491)

Characteristic	HEWS ≥ 3 (*n* = 3181, 57.9%)	HEWS ≥ 5 (*n* = 2578, 46.9%)	NEWS2 ≥ 5 (*n* = 3416, 62.2%)
Sensitivity (95% CI)	95.9 (94.9–96.7)	75.9 (73.9–77.9)	84.5 (82.8–86.2)
Specificity (95% CI)	44.6 (42.9–46.2)	67.6 (66.1–69.1)	49.0 (47.4–50.7)
Positive predictive value (95% CI)	46.5 (45.7–47.2)	54.1 (52.8–55.4)	45.5 (44.5–46.4)
Negative predictive value (9% CI)	95.5 (94.5–96.4)	84.8 (83.7–85.9)	86.3 (84.9–87.6)
Positive likelihood ratio (95% CI)	1.73 (1.68–1.78)	2.35 (2.22–2.47)	1.66 (1.60–1.72)
Negative likelihood ratio (95% CI)	0.09 (0.07–0.12)	0.36 (0.33–0.39)	0.32 (0.28–0.35)
Number needed to examine (95% CI)	2.15 (2.12–2.18)	1.85 (1.81–1.89)	2.20 (2.16–2.25)

*CI* confidence interval, *HEWS* Hamilton Early Warning Score, *NEWS2* National Early Warning Score 2

Table 4 describes the prognostic accuracy of HEWS and NEWS2 among the cohort of patients with suspected infection. A HEWS ≥ 5 was found among 799 patients (46.8%). Sensitivity of HEWS ≥ 5 was 83.2% (95% CI 79.8–86.1), and specificity was 71.4% (68.7–74.1). The NNE was 1.69. Conversely, 1075 patients with suspected infection (62.9%) had a NEWS2 ≥ 5. Sensitivity was 87.7% (95% CI 84.7–90.3), and specificity was 49.1% (95% CI 46.2–52.1). The NNE was 2.19 (95% CI 2.11–2.27). Among patients with suspected infection, the rTPR of NEWS2 to HEWS was 1.05 (95% CI 0.94–1.17, *P* = 0.38) and the rFPR was 1.78 (95% CI 1.66–1.89, *P* < 0.01).

**Table 4:** Prognostic accuracy of HEWS and NEWS2 for in-hospital mortality—patients with suspected infection (*n* = 1708)

Characteristics	HEWS ≥ 3 (*n* = 1176, 68.9%)	HEWS ≥ 5 (*n* = 799, 46.8%)	NEWS2 ≥ 5 (*n* = 1075, 62.9%)
Sensitivity (95% CI)	96.0 (94.0–97.4)	83.2 (79.8–86.1)	87.7 (84.7–90.3)
Specificity (95% CI)	44.7 (41.8–47.7)	71.4 (68.7–74.1)	49.1 (46.2–52.1)
Positive predictive value (95% CI)	46.5 (45.2–47.9)	59.3 (56.9–61.7)	45.7 (44.1–47.3)
Negative predictive value (95% CI)	61.8 (59.5–64.1)	89.4 (87.6–91.1)	89.1 (86.7–91.1)
Positive likelihood ratio (95% CI)	1.74 (1.64–1.83)	2.91 (2.64–3.21)	1.72 (1.62–1.84)
Negative likelihood ratio (95% CI)	0.09 (0.06–0.14)	0.24 (0.20–0.28)	0.25 (0.20–0.32)
Number needed to examine (95% CI)	2.15 (2.09–2.21)	1.69 (1.62–1.76)	2.19 (2.11–2.27)

*CI* confidence interval; *HEWS* Hamilton Early Warning Score, *NEWS2* National Early Warning Score 2

**Fig. 1:**
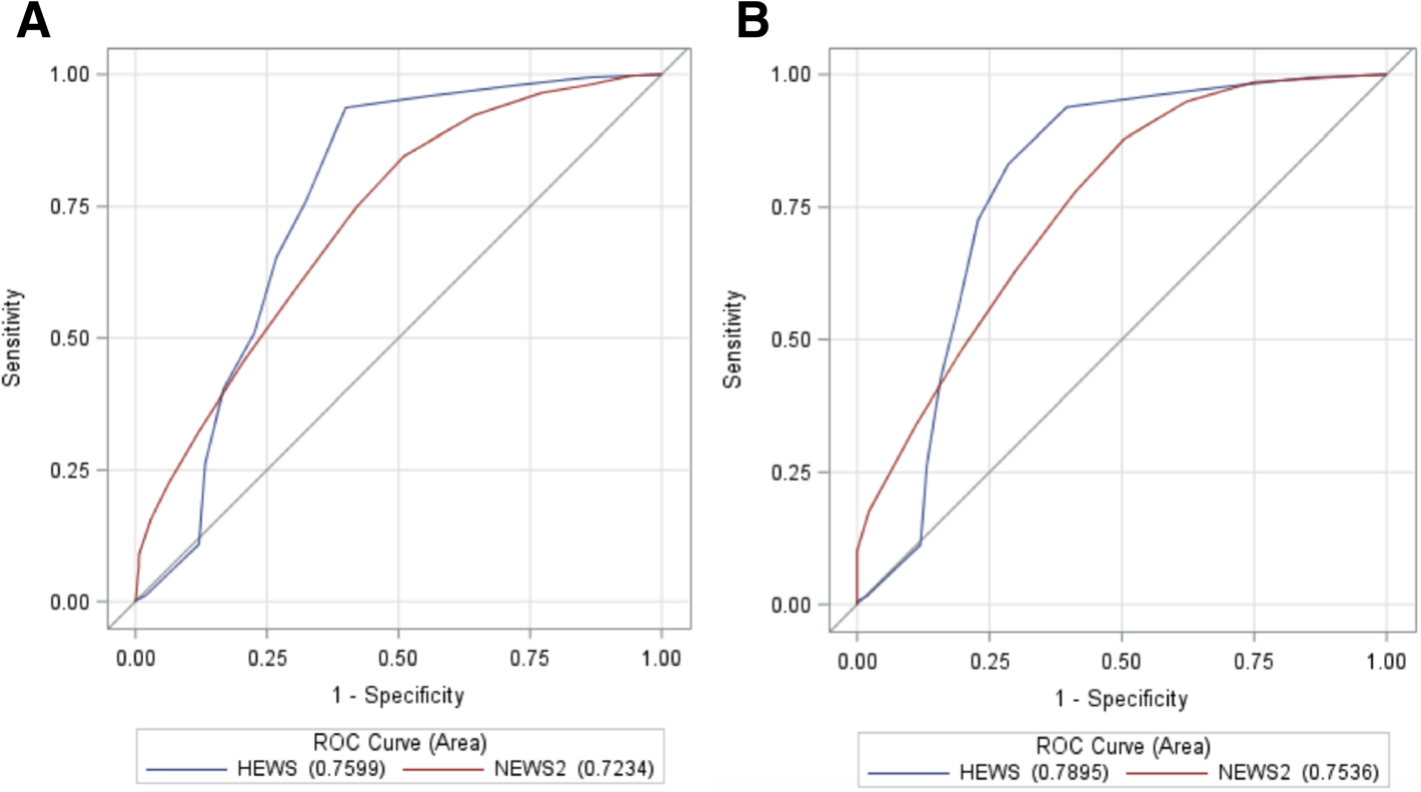
Receiver operating characteristic curves for prediction of mortality. **a** Entire cohort of RRT patients (*n* = 5491). **b** Patients with suspected infection (*n* = 1708)

AUROC curves of HEWS and NEWS2 for prediction of in-hospital mortality among the entire RRT cohort and those only with suspected infection are displayed in Fig. 1. Among the entire RRT cohort (Fig. 1a), HEWS had an AUROC of 0.76 (95% CI 0.75–0.77) and NEWS2 had an AUROC of 0.72 (95% CI 0.71–0.74). For patients with suspected infection only (Fig. 1b), HEWS had an AUROC of 0.79 (95% CI 0.77–0.81), while NEWS2 had an AUROC of 0.75 (95% CI 0.73–0.78).

## Discussion

We evaluated the prognostic accuracy of the HEWS and the NEWS2 among hospitalized patients with acute deterioration, requiring RRT assessment. We found that both HEWS and NEWS2 accurately predicted in-hospital mortality in this population and particularly among patients with suspected infection. At the critical threshold (≥ 5), the NEWS2 had comparable sensitivity to HEWS (as evidenced by rTPR), but lower specificity (as evidenced by rFPR). This was true among the entire cohort, as well as those patients with suspected infection. Taken together, this work provides novel evidence comparing the prognostic accuracy of the NEWS2 and HEWS scores in a population of hospitalized patients at high risk for deterioration.

RRTs serve an important role in the hospital Early Warning System. While RRT intervention can assist with end-of-life care and reduce the rate of unexpected death, it also functions to aid in the care of hospitalized patients outside of the ICU, in order to optimize their management and disposition. To that end, providers often have to prognosticate for risk of future deterioration in order to guide RRT resources. Among hospitalized patients, risk of deterioration can often be predicted through vital signs [28]. However, despite this, there can be delay to RRT activation, and a patient may deteriorate further. Furthermore, certain populations of patients (such as the elderly) may deteriorate prior to objective changes in vital signs, which may contribute to delay to RRT activation [29]. Delays in RRT activation are common and also often reflect variation in time of day, personnel on duty, and patient volume [30, 31]. Prolonged delays are associated with increased mortality in this population [32]. For this reason, EWSs with mandatory calling criteria have an important role in the identification of deteriorating patients and the activation of the RRT.

Incorporating EWSs into electronic ward and health databases provides a promising method for the immediate detection of patients who may deteriorate [33]. However, at present, the ideal utility of an EWS is unknown. It may seem optimal for an EWS to maximize sensitivity, and thereby reduce the number of false negatives. However, in practice, an EWS that is highly sensitive but poorly specific may result in unnecessary workload, alarm fatigue, and an inability of RRT or ICU capacity to meet demand [8]. For this reason, experts have endorsed the utilization of the NNE metric, as it serves to quantify the trade-off between an EWS that is poorly sensitive and thus misses many deteriorating patients, and an EWS that is poorly specific, resulting in frequent activation and alarm fatigue [27].

In this study, we found that the NEWS2 score had comparable sensitivity to HEWS (84.5% vs. 75.9%, rTPR 1.11). However, the specificity of NEWS2 was poorer (49.0%), as compared to HEWS (67.6%), with a rFPR of 1.57. When comparing the NNE, HEWS was superior to NEWS2 (1.85 vs. 2.20). Until this point, there has been little external validation of the NEWS2 score [12]. Overall, we found that the specificity of HEWS (as evidence by rFPR) and its lower NNE may suggest that it may be the preferable EWS in reducing alarm fatigue.

Deterioration among patients admitted to the hospital wards with suspected infection is especially common [15]. Such patients are at risk of developing sepsis and septic shock, unplanned ICU admission, and death [15]. Therefore, significant effort has been directed at the early identification of deterioration in this population. Traditionally, the SIRS criteria have been used for this purpose [34, 35]. Our group has previously shown that, while the SIRS criteria has demonstrated high sensitivity among RRT patients with suspected infection, this has come at the cost of poor specificity [19]. In contrast, while qSOFA [17, 18] has been associated with high specificity for mortality among RRT patients with suspected infection, its overall sensitivity is poor [19, 36]. Churpek et al. evaluated the prognostic accuracy of qSOFA against other EWSs, such as the original NEWS and the Modified Early Warning Score (MEWS), and found that qSOFA was inferior to these existing EWSs in predicting in-hospital mortality [37]. We found that the HEWS score had superior sensitivity and NNE than qSOFA, when applied to our population of RRT patients with suspected infection. This suggests a possible benefit of utilizing the HEWS score as compared to qSOFA among RRT patients with suspected infection, as the higher sensitivity of HEWS may function as a better prompt for escalating treatment.

This study has several strengths, including a large sample size, comparisons among patients with and without suspected infection, and comprehensive data on many patient and RRT variables. Furthermore, our study provides one of the first external applications of both the HEWS and NEWS2. However, there are several limitations that hinder the generalizability of our data. Most importantly, our study does not address the question related to EWS accuracy in identification of patients prior to deterioration, as we only included patients who had experienced RRT activation. We still sought to investigate the prognostic accuracy of these tools for in-hospital mortality and ICU admission, as it provides evidence for the use of HEWS and NEWS2 in risk-stratification among RRT-activated patients. Future prospective work should focus on HEWS and NEWS2 accuracy in identifying patients at risk of deterioration prior to RRT activation. Second, while our database included consecutive patients, NEWS2 and HEWS scores were gathered on the basis of the patient’s first RRT call. Therefore, it is possible that patients may have initially had a lower NEWS2 or HEWS score on initial assessment, but then subsequently deteriorated and died in-hospital on a later date. Third, while we utilized the NNE as a metric of EWS effectiveness, it is important to note that the NNE is derived from the positive predictive value, and therefore is influenced by the prevalence of the studied outcome. Thus, these NNE values may not be generalizable to populations with lower mortality rates. Additionally, 8.7% of patients in our original cohort were excluded due to insufficient data, though the mortality of this population did not differ from our included patients. Finally, while our data were gathered from different centers, they exist within the same health network and city and therefore may be susceptible to local variation in ICU admission and treatment practices.

## Conclusion

We found that that the HEWS and NEWS2 had similar overall prognostic accuracy for prediction of in-hospital mortality among RRT patients, and particularly those with suspected infection. While a NEWS2 score ≥ 5 had comparable sensitivity for mortality as a HEWS score ≥ 5, this came at the cost of much lower specificity and a higher overall NNE and therefore may contribute to alarm fatigue. Knowledge related to the prognostic performance of these two EWSs will allow RRT clinicians to properly contextualize their use in the care of patients with acute deterioration.

## Additional files

Additional file 1: **Table S1.** Rapid response team criteria at The Ottawa Hospital. Rapid response team criteria. (DOCX 101 kb)

Additional file 2: **Figure S1.** Comparison of the HEWS and NEWS2 Scores. Comparison of the HEWS and NEWS2 Scores. (DOCX 179 kb)

Additional file 3: **Figure S2.** Study flow diagram. (DOCX 56 kb)

Additional file 4: **Table S2.** Prognostic accuracy of HEWS and NEWS2 for ICU admission. Prognostic accuracy of HEWS and NEWS2 for ICU admission. (DOCX 65 kb)

## Abbreviations

AUROC: Area under the receiver operating characteristics curve
CI: Confidence interval
EWS: Early Warning Score
HEWS: Hamilton Early Warning Score
ICU: Intensive care unit
IQR: Interquartile range
LOS: Length of stay
NEWS2: National Early Warning Score 2
NNE: Number needed to examine
qSOFA: Quick Sequential (Sepsis-related) Organ Failure Assessment
rFPR: Relative false-positive rate
RRT: Rapid response team
rTPR: Relative true-positive rate
SD: Standard deviation
SIRS: Systemic inflammatory response syndrome
