# Supplementary material for: Prognostic accuracy of the Hamilton Early Warning Score (HEWS) and the National Early Warning Score 2 (NEWS2) among hospitalized patients assessed by a rapid response team
Source: Crit Care. 2019 Feb 21;23:60. doi: 10.1186/s13054-019-2355-3 (PMC6385382; doi:10.1186/s13054-019-2355-3)
Supplement: Supplementary file 1 — Table S1. Rapid response team criteria at The Ottawa Hospital. Rapid response team criteria. (DOCX 101 kb) [file 13054_2019_2355_MOESM1_ESM.docx]

**Supplemental Figure 1** – Comparison of the HEWS and NEWS2 Scores. Abbreviations: A = alert; CAM+ = Confusion assessment method (CAM) screening possible for potential delirium; CNS = central nervous system; P = pain; U = unresponsive; V = voice.

**Hamilton Early Warning Score (HEWS)**

| **Physiologic Parameters** | **3** | **2** | **1** | **0** | **1** | **2** | **3** |
| --- | --- | --- | --- | --- | --- | --- | --- |
| Heart Rate |  | ≤40 | 41-50 | 51-100 | 101-110 | 111-130 | >130 |
| Systolic Blood Pressure | <71 | 71-90 |  | 91-170 |  | 171-200 | >200 |
| Temperature | ≤35 |  | 35.1-36 | 36.1-37.9 | 38-39 | ≥39.1 |  |
| Respiratory Rate | <8 | 8-13 |  | 14-20 |  | 21-30 | >30 |
| Oxygen Saturation | <85 |  | 85-92 | >92 |  |  |  |
| Supplemental Oxygen |  |  |  | Room air | ≤5 L/min |  | >5L/min |
| CNS Change from Baseline |  | CAM+ |  | A | V | P | U |

**National Early Warning Score 2 (NEWS2)**

| **Physiologic Parameters** | **3** | **2** | **1** | **0** | **1** | **2** | **3** |
| --- | --- | --- | --- | --- | --- | --- | --- |
| Respiratory Rate | <8 |  | 9-11 | 12-20 |  | 21-24 | ≥25 |
| SpO2 Scale 1 (%) | ≤91 | 92-93 | 94-95 | ≥96 |  |  |  |
| SpO2 Scale 2 (%) | ≤83 | 84-85 | 86-87 | 88-92  ≥93 on air | 93-94 on oxygen | 95-96 on oxygen | ≥97 on oxygen |
| Air or oxygen? |  | Oxygen |  | Air |  |  |  |
| Systolic Blood Pressure | ≤90 | 91-100 | 101-110 | 111-219 |  |  | ≥220 |
| Heart Rate | ≤40 |  | 41-50 | 51-90 | 92-110 | 111-130 | ≥131 |
| Consciousness |  |  |  | Alert |  |  | AVPU |
| Temperature | ≤35.0 |  | 35.1-36.0 | 36.1-38.0 | 38.1-39.0 | ≥39.1 |  |
