# Supplementary material for: Prognostic accuracy of the Hamilton Early Warning Score (HEWS) and the National Early Warning Score 2 (NEWS2) among hospitalized patients assessed by a rapid response team
Source: Crit Care. 2019 Feb 21;23:60. doi: 10.1186/s13054-019-2355-3 (PMC6385382; doi:10.1186/s13054-019-2355-3)
Supplement: Supplementary file 2 — Figure S1. Comparison of the HEWS and NEWS2 Scores. Comparison of the HEWS and NEWS2 Scores. (DOCX 179 kb) [file 13054_2019_2355_MOESM2_ESM.docx]

**Supplemental Figure 2** – Study Flow Diagram. Abbreviations: HEWS = Hamilton Early Warning Score; NEWS2 = National Early Warning Score 2; RRT = Rapid Response Team

**
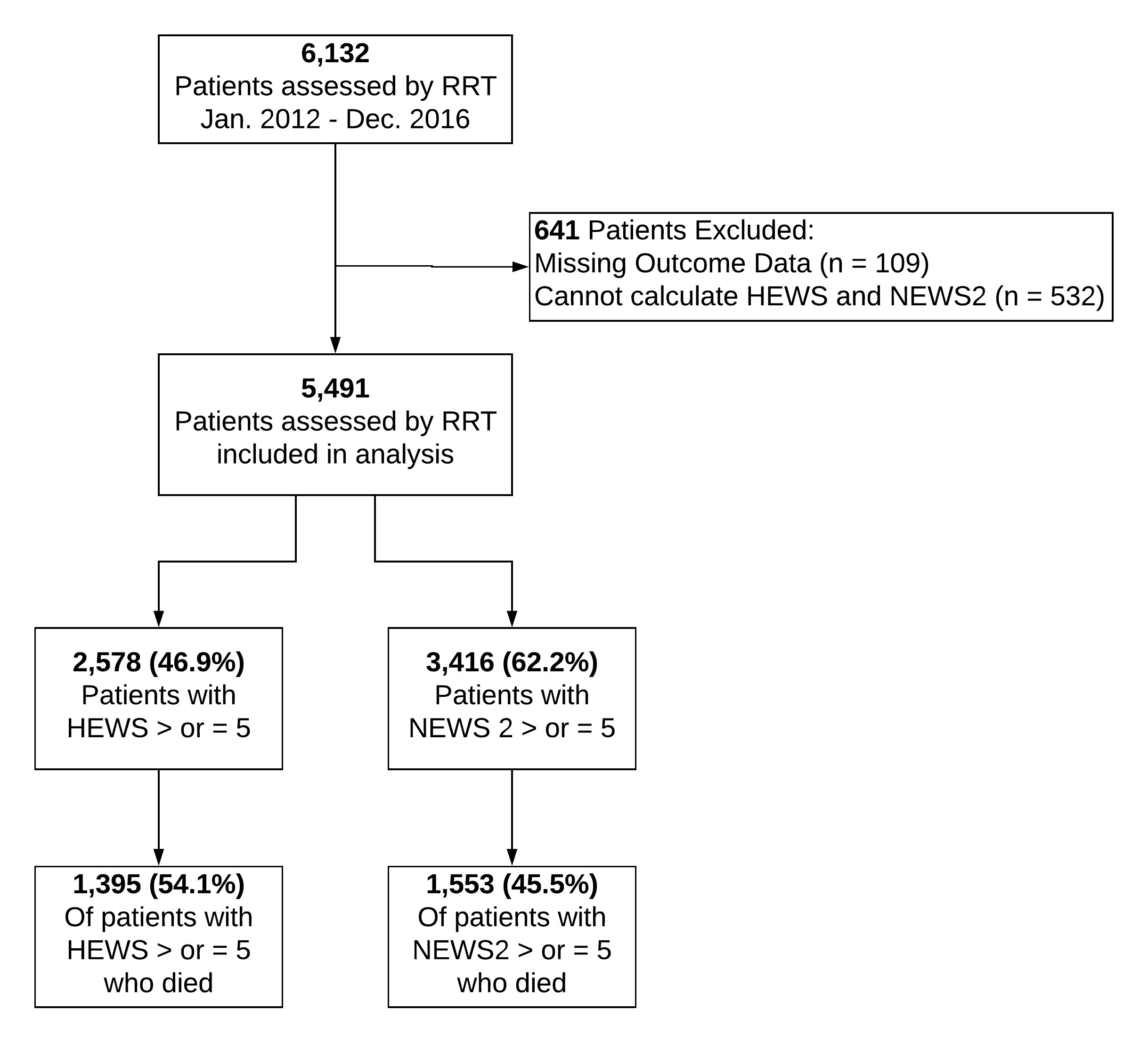
**
