# Supplementary material for: Prognostic accuracy of the Hamilton Early Warning Score (HEWS) and the National Early Warning Score 2 (NEWS2) among hospitalized patients assessed by a rapid response team
Source: Crit Care. 2019 Feb 21;23:60. doi: 10.1186/s13054-019-2355-3 (PMC6385382; doi:10.1186/s13054-019-2355-3)
Supplement: Supplementary file 3 — Figure S2. Study flow diagram. (DOCX 56 kb) [file 13054_2019_2355_MOESM3_ESM.docx]

**Supplemental Table 1:** Rapid Response Team criteria at The Ottawa Hospital.

| **Airway** | Threatened stridor; excessive secretions |
| --- | --- |
| **Breathing** | Respiratory rate ≤ 8 breaths/minute or ≥ 30 breaths/minute |
| **Circulation** | Systolic blood pressure ≤ 90 mmHg or ≥ 200 mmHg or ≥ 40 mmHg decrease  Heart rate ≤ 40 beats/minute or ≥ 130 beats/minute |
| **Level of Consciousness** | >2 point decrease in Glasgow Coma Scale |
| **Oxygen Saturation** | <90% on 50% FiO_2_ or 6 litres/minute |
| **Urine Output** | <100 mL over four hours |
| **Other** | Health care worker “worried” about the patient, needs medical assistance, failure to respond to treatment |
