# Supplementary material for: Prognostic accuracy of the Hamilton Early Warning Score (HEWS) and the National Early Warning Score 2 (NEWS2) among hospitalized patients assessed by a rapid response team
Source: Crit Care. 2019 Feb 21;23:60. doi: 10.1186/s13054-019-2355-3 (PMC6385382; doi:10.1186/s13054-019-2355-3)
Supplement: Supplementary file 4 — Table S2. Prognostic accuracy of HEWS and NEWS2 for ICU admission. Prognostic accuracy of HEWS and NEWS2 for ICU admission. (DOCX 65 kb) [file 13054_2019_2355_MOESM4_ESM.docx]

**Supplemental Table 2** – Prognostic Accuracy of HEWS and NEWS2 for ICU Admission – Entire Cohort of RRT Patients, only including those with Limits of Care Allowing for ICU Admission (*n* = 4,404). Abbreviations: CI = Confidence Interval; HEWS = Hamilton Early Warning Score; NEWS2 = National Early Warning Score 2.

| **Characteristic** | **HEWS ≥ 3**  (*n* = 2418, 54.9%) | **HEWS ≥ 5**  (*n* = 2046, 46.5%) | **NEWS2 ≥ 5**  (*n* = 2262, 51.3%) |
| --- | --- | --- | --- |
| Sensitivity (95% CI) | 91.8 (90.2-93.1) | 76.4 (74.1-78.6) | 83.4 (81.4-85.3) |
| Specificity (95% CI) | 63.3 (61.6-65.1) | 68.4 (66.6-70.0) | 64.5 (62.7-66.2) |
| Positive Predictive Value (95% CI) | 55.3 (54.1-56.5) | 54.4 (52.9-55.9) | 53.7 (52.4-55.1) |
| Negative Predictive Value (95% CI) | 94.0 (92.9-94.9) | 85.4 (84.2-86.6) | 88.7 (87.5-89.8) |
| Positive Likelihood Ratio (95% CI) | 2.50 (2.38-2.63) | 2.41 (2.27-2.56) | 2.35 (2.22-2.48) |
| Negative Likelihood Ratio (95% CI) | 0.13 (0.11-0.15) | 0.35 (0.31-0.38) | 0.26 (0.23-0.29) |
| Number Needed to Examine (95% CI) | 1.81 (1.76-1.85) | 1.84 (1.79-1.89) | 1.86 (1.81-1.91) |
